# Mouse Models of Food Allergy in the Pursuit of Novel Treatment Modalities

**DOI:** 10.3389/falgy.2021.810067

**Published:** 2021-12-15

**Authors:** Johanna M. Smeekens, Michael D. Kulis

**Affiliations:** ^1^Division of Allergy and Immunology, Department of Pediatrics, University of North Carolina School of Medicine, Chapel Hill, NC, United States; ^2^University of North Carolina Food Allergy Initiative, Chapel Hill, NC, United States

**Keywords:** food allergy, peanut allergy, mouse models, microbiome, immunotherapy

## Abstract

The prevalence of IgE-mediated food allergies has increased dramatically in the past three decades, now affecting up to 10% of the US population. IgE-mediated food allergy is an immunologic disease, involving a variety of cells, including B and T cells, mast cells, basophils, ILC2s, and epithelial cells. Mouse models of food allergy mimic the overall immunologic processes known to exist in humans. Due to the limitations of invasive sampling of human tissue and the similarities of the human and mouse immune systems, comprehensive pathogenesis studies of food allergy have been performed in mouse models. Mouse models have been effective in elucidating the roles of non-oral routes of sensitization and identifying key cells and molecules involved in allergic sensitization. Furthermore, the development of novel therapeutic approaches for food allergy has been accelerated through the use of pre-clinical mouse models. Despite the groundbreaking findings stemming from research in mice, there are continued efforts to improve the translational utility of these models. Here, we highlight the achievements in understanding food allergy development and efforts to bring novel treatment approaches into clinical trials.

## Introduction

IgE-mediated food allergies now affect an estimated 10% of the US population (1), which is a substantial increase from estimates generated over 20 years ago (2). The most common food allergies in the US are milk, egg, peanut, tree nuts, soy, wheat, fish, shellfish, and sesame. Reactions to accidental exposures are common and can be severe (3). Although fatal reactions brought on by allergic reactions to foods are exceedingly rare, they do happen (4). It is this uncertainty that causes anxiety and quality of life impairment in those with food allergies and their caregivers (5). Additionally, health care costs for food allergies in the US are approximately $25 billion annually (6). Taken together, food allergy represents a significant public health concern.

**Figure 1:**
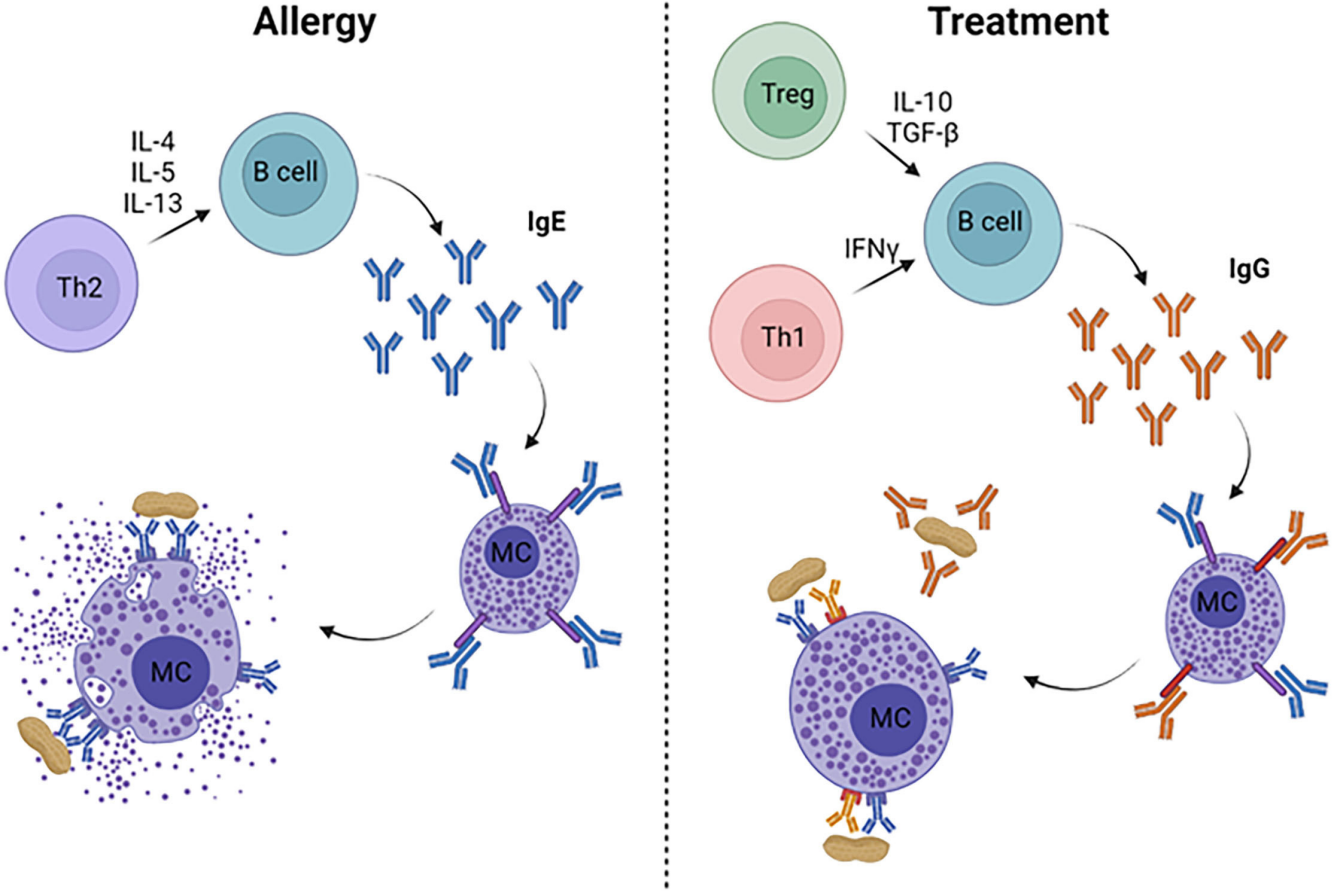
Immune mechanisms of allergy and its treatment. Food allergy is characterized by a Th2-dominated response, leading to production of allergen-specific IgE from B cells. IgE binds to FcεRI on mast cells and basophils (not pictured), which degranulate when surface-bound IgE is cross-linked, resulting in allergic symptoms and anaphylaxis. Successful treatments lead to Th1- and/or Treg-skewed immune responses that drive high levels of allergen-specific IgG, which can inhibit mast cell degranulation through allergen neutralization and/or binding to the inhibitory FcγRIIb on mast cells and basophils.

Food allergies are an immunologic disease characterized by a Th2-driven response, resulting in the production of allergen-specific IgE (7). The current paradigm is that an initial sensitizing event occurs at an epithelial surface (i.e., skin, airway, gastrointestinal tract) where the food antigens come into contact with antigen presenting cells (APCs) (8). Once taken up by APCs, the antigens are processed and displayed through MHC class II molecules that allow activation of naïve T cells. The T cell fate is driven by the local cytokine milieu, now understood to be Th2-promoting cytokines from ILC2 cells and epithelial-derived cytokines such as TSLP, IL-33, and IL-25. In the presence of these pro-Th2 cytokines, the naïve T cells undergo differentiation into Th2-type cells, which then interact with B cells to cause class-switching into IgE-producing B cells (Figure 1). Ultimately, these B cells become long-lived plasma cells that may produce allergen-specific IgE for many years. Once IgE is in circulation, it binds with high affinity to FcεRI receptors on mast cells and basophils, priming these effector cells for allergic reactions. Specifically, subsequent exposures to allergenic foods will cross-link IgE on mast cells and basophils causing a signaling cascade that ends in degranulation and release of allergic mediators, such as histamine, leukotrienes, prostaglandins, and cytokines. These mediators result in the symptoms seen during an allergic reaction, including mild symptoms such as urticaria, emesis, diarrhea, edema, and more severe symptoms which include hypotension, neurologic compromise, and cardiovascular collapse.

Our understanding of the immunobiology of human food allergy has several limitations. For example, the sensitization phase in humans is extraordinarily difficult to study since patients typically present to an allergist after they've already experienced a food-induced reaction. This makes it impossible to pinpoint the sensitization event and what environmental factors may have led to the production of IgE. Other limitations in studying the human disease are the types of samples that can be acquired, often restricted to blood, saliva, urine, and stool. Conducting biopsies of skin and gastrointestinal (GI) tissue may be helpful, although these procedures are not routinely done on infants with food allergy. Our understanding of anaphylaxis is also limited because of the ethical constraints associated with intentionally causing moderate to severe allergic reactions. For these reasons, research has relied on animal models to better understand the pathophysiology of food allergy. In particular, mouse models have become a mainstay in food allergy research.

Mouse models of food allergy mimic key factors of the human disease (9). Upon sensitization to foods, mice will produce allergen-specific IgE, Th2-type cytokines, and will undergo mast cell-induced anaphylaxis upon food challenge. Importantly, we can also control the sensitizing events in mice, as well as the environmental and genetic factors that may drive food allergy. Collection of lymph nodes, spleens, and GI tissue, which are not studied in humans, has also furthered our understanding of food allergies. Finally, mouse models have shed light on mediators involved in anaphylaxis, such as platelet activating factor (PAF) (10). Based on an improved understanding of what causes food allergies and the molecules involved in anaphylaxis, we may be able to develop targeted prevention and therapeutic approaches. Indeed, mouse models have become an important pre-clinical tool to test novel therapies. In this review, we will focus on findings from mouse models that encompass the progress made in understanding immunologic mechanisms underlying food allergy and therapeutic strategies for their treatment.

## Modeling Sensitization Through Various Exposure Routes

### Oral and Systemic Sensitization

As in humans, feeding food antigens orally to mice generally leads to the induction of oral tolerance (11). To circumvent tolerance mechanisms, researchers have turned to adjuvants to skew the immune response toward Th2. Classically, models of food allergy have relied on Th2-skewing adjuvants to induce sensitization. The most commonly used Th2-adjuvants are cholera toxin (CT), staphylococcal enterotoxin B (SEB), and aluminum hydroxide (alum). CT and SEB are co-administered with food proteins through the GI tract (12, 13), while allergens adsorbed to alum are delivered via intraperitoneal injection (14). The use of these adjuvants have led to models of allergy to peanut, milk, egg, tree nuts, shellfish, among others, in BALB/c, C3H/HeJ and C57BL/6 mice, demonstrated by the production of allergen-specific IgE, Th2 cytokines and anaphylaxis upon food challenge (9). There are two strains of mice, CC027/GeniUnc and Il4raF709, that can be enterally sensitized to food antigens in the absence of adjuvant. The CC027/GeniUnc mice come from the Collaborative Cross, and are an inbred strain resulting from funnel breeding of eight founder strains (15, 16). We have recently demonstrated that CC027/GeniUnc mice become allergic to peanut and walnut without a Th2-skewing adjuvant, and sensitization is associated with increased gut permeability, decreased fecal IgA and a unique gut microbiome (17). The Il4raF709 mice, in which the IL-4Rα immunoreceptor tyrosine-based inhibitory motif is inactivated, can be sensitized to OVA in the absence of adjuvant (18). Interestingly, these mice also have increased gut permeability, suggesting that epithelial disruption may be a key driving force to orally-induced food allergy.

### Epicutaneous Sensitization

As the paradigm for sensitization in humans has shifted to non-oral routes, animal models have been employed to help characterize these alternate routes of exposure. An impaired skin barrier in humans resulting from eczema or filaggrin mutations is associated with increased prevalence of food allergy. Mouse models have been developed that mimic eczema, including flaky tail (filaggrin deficient) mice (19) and in BALB/c mice with tape stripping (20), which removes the stratum corneum layer of the skin. Food antigens are applied to the impaired skin several times over the course of a few weeks, which leads to allergic sensitization. Importantly, in the flaky tail model, proteolytic allergens are co-administered with peanut to serve as an adjuvant. More recently, mice were sensitized to peanut through the skin in the absence of adjuvant or impaired skin, but milk or egg allergens administered on the skin did not lead to sensitization, indicating an inherent adjuvant property of peanut (21). Indeed, milk allergen applied to the skin with Ara h 2 led to induction of milk-specific IgE. Overall, data from these animal models provide convincing evidence that sensitization through the skin can lead to food allergy.

### Airway Sensitization

Another potential non-oral route of exposure leading to sensitization is the airway, although human data is limited. Mouse models have demonstrated that sensitization to peanut can occur through the airway. Our group demonstrated that airway exposure to peanut plus house dust leads to the induction of peanut-specific IgE and anaphylaxis upon peanut challenge, indicating that house dust acts as an adjuvant to induce airway sensitization (22). Further studies of this model have demonstrated that the adjuvant activity is dependent upon MyD88, and co-administration of TLR signaling molecules, LPS, CpG, flagellin, PAM with peanut induces allergy (23). Using a different model, airway sensitization to peanut was demonstrated in the absence of an adjuvant. This model relied on airway exposure to peanut flour to produce peanut-specific IgE (24). Interestingly, both of these airway exposure models identified a role for Tfh cells in the induction of peanut allergy.

### Humanized Models

Humanized mouse models of food allergy have also been reported. These models are attractive since they utilize human B and T cells, leading to the *in vivo* production of allergen-specific human IgE. In one model, CD34^+^ human hematopoietic stem cells were transferred into NSG mice and became successfully engrafted after 4 months (25). Human CD45^+^ cells were found in peripheral blood and tissues, with CD4^+^ T cells and CD19^+^ B cells found in the spleen. Furthermore, mast cells were identified in the intestinal mucosa. After engraftment, mice were orally exposed to peanut for 8 weeks, leading to production of peanut-specific IgE and anaphylaxis on peanut challenge. A second example of a humanized mouse model used PBMCs isolated from peripheral blood combined with peanut antigens to reconstitute NSG mice (26). Subsequently, mice were exposed to peanut for an additional 5 weeks to induce peanut-specific IgE production. These models highlight the possibility of studying food allergy *in vivo* using human cells.

### Limitations

Despite the many advantages of mouse models, there are several limitations. One major difference between mouse and human sensitization is that anaphylaxis is not solely IgE-dependent in mice, due to the production of anaphylactic IgG1. This was definitively demonstrated in mice lacking IgE, which experienced anaphylaxis upon antigen challenge (27). Another key limitation is the cells involved in anaphylaxis differ in humans in mice. In humans, food-induced anaphylaxis is driven by mast cells and basophils, whereas in mice, mast cells, basophils, neutrophils and macrophages have all been shown to play an important role (28–30). While humanized mouse models may more closely mimic the human disease, these models are currently hampered by the requirement of access to human blood, a substantially longer peanut allergy induction time, and higher costs. These limitations indicate further research is needed to develop optimal mouse models of food allergy.

## Therapeutic Approaches

In addition to understanding how sensitization occurs, another powerful use of mouse models is developing novel therapies. Pre-clinical safety and efficacy studies in mice are necessary to bring treatments forward into Phase 1 clinical trials. In this section, we will review antigen-specific and non-specific therapeutic approaches and their mechanisms (Figure 1).

### Pre-clinical Therapies That Led to Clinical Trials

There are several examples of therapies developed in mouse models that have reached clinical trials: epicutaneous immunotherapy (EPIT), mutated peanut allergens expressed in *E. coli* (EMP-123), and food allergy herbal formula (FAHF). EPIT for peanut allergy was initially tested in BALB/c mice using a Viaskin peanut patch. This approach used electrostatically sprayed peanut protein on a patch applied to intact skin. Application of the patch creates a condensation chamber where peanut protein is solubilized and absorbed into the epidermis. In pre-clinical mouse studies, patch application led to an immunomodulatory effect highlighted by increased peanut-specific IgG2a (31, 32). Later studies found increased LAP^+^ Tregs in the GI tract of mice treated with EPIT, indicating a skin-gut axis mechanism (33). EPIT was tested in humans, in Phase 1, 2, and 3 clinical trials. These trials demonstrated an extremely favorable safety profile, although efficacy was limited to 35% of the study population after 1 year of treatment in the Phase 3 trial (34).

Another example is mutated Ara h 1, 2, and 3 expressed in *E. coli*. The premise of this treatment was to introduce mutations into the IgE binding epitopes of the major peanut allergens Ara h 1, 2, and 3 to reduce IgE binding capacity and mast cell degranulation, while retaining their ability to stimulate peanut-specific T cells. Expression in heat/phenol-killed *E. coli* was found to be an effective delivery system when given rectally in mice. In C3H/HeJ mice, this form of therapy led to increased peanut-specific IgG2a, decreased Th2-type cytokines, increased Tregs, and ultimately prevented reactions to peanut upon oral challenge (35). Finally, a Phase 1 clinical trial was undertaken in 10 peanut-allergic subjects to investigate the safety of this therapy. Unfortunately, the majority of subjects experienced symptoms, with half of the subjects having frequent adverse reactions, including 20% that experienced anaphylaxis, preventing completion of the protocol (36).

A non-antigen specific approach to treating food allergy arose from Traditional Chinese Medicine. The food allergy herbal formula (FAHF-2) is a blend of nine herbs given orally over the course of several weeks. In a C3H/HeJ model of peanut allergy, FAHF-2 blocked anaphylaxis and led to decreased peanut-specific IgE and Th2-type cytokines, with increased peanut-specific IgG2a and Th1-type cytokine production (37). FAHF-2 was tested for safety in a Phase 1 clinical trial and was found to be well-tolerated (38). In the Phase 2 trial, subjects took 10 tablets three times a day for 6 months, and there was no improvement in food challenge outcomes in the active or placebo arms, and no differences between arms in peanut-specific IgE, IgG4, IL-5, IL-13, IL-10, and IFN-γ (39).

### Therapies Targeting T Cells

There are several approaches that have shown promise in pre-clinical mouse models but have not yet made it into clinical trials. Since T cells are important in driving food allergy, therapies to suppress Th2 responses may be effective. One modality using peptides from the major egg allergen ovomucoid were shown to stimulate T cells, but lacked the ability to cross-link IgE, therefore rendering them unable to cause allergic reactions. When delivered orally for 4 weeks, this peptide-based immunotherapy induced Tregs and ovomucoid-specific IgA, while reducing ovomucoid-specific IgE, ultimately preventing anaphylaxis (40). Another approach used a whole peanut extract (WPE) linked to syngeneic splenocytes (SP) to induce antigen-specific immune tolerance (41). In a prophylactic model, i.v. administration of WPE-SP completely prevented production of peanut-specific IgE and led to minimal Th2 cytokine production. When applied in a therapeutic mouse model, two doses of WPE-SP led to significant suppression of anaphylaxis upon challenge, which was associated with decreased peanut-specific IgE and peanut-induced Th2 cytokines. Modulating T cell responses through the use of Th1-skewing adjuvants is another therapeutic approach. The TLR9 ligand, CpG, has been demonstrated to prevent and treat peanut allergy when given orally, intranasally, or by intraperitoneal injection. These studies all demonstrate the ability of CpG to induce IFN-γ responses from peanut-specific T cells, with a subsequent increase in peanut-specific IgG2a (42–45). Finally, since virus-like particles (VLPs) are known to modulate CD4^+^ T cells, VLPs displaying food allergens may alter T cell responses leading to the production of allergen-specific IgG (46). Indeed, VLPs displaying Ara h 1 and 2 induced large quantities of allergen-specific IgG, which were protective against allergen challenge (47).

### Therapies Modulating the Gut Microbiome

**Figure 2:**
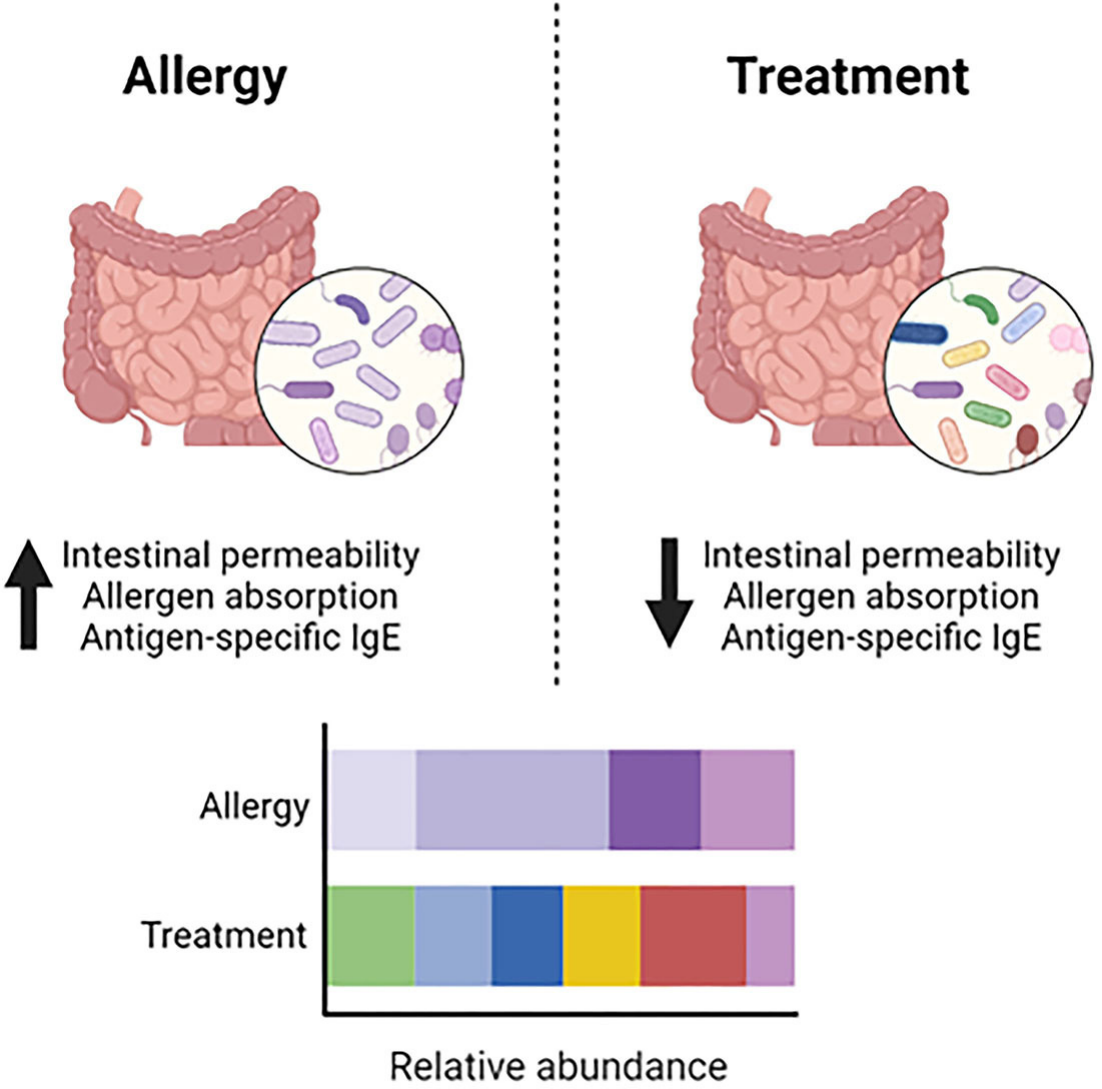
Dysbiosis and modulation of the gut microbiome. In food allergy, dysbiosis in the gut microbiome contributes to increased intestinal permeability, allergen absorption and production of antigen-specific IgE. Modulating the gut microbiome to increase microbial diversity and the abundance of beneficial bacteria leads to decreased intestinal permeability, allergen absorption and production of antigen-specific IgE.

Studies have demonstrated the importance of the gut microbiome in food allergy in humans and mice (48). One study demonstrated that mice treated with antibiotics and then sensitized to peanut plus cholera toxin produced significantly higher peanut-specific IgE and IgG1 compared to non-antibiotic treated mice (49). Additionally, in germ-free mice, devoid of enteral microbes, oral food protein exposure led to elevated IgE and anaphylaxis upon oral challenge (49). Therefore, modulating the gut microbiome *via* the addition of protective bacteria or suppression of pathogenic bacteria may protect against various food allergies. Gnotobiotic mice reconstituted with Clostridia and sensitized with peanut plus cholera toxin had reduced peanut-specific IgE compared to germ-free controls. Furthermore, Clostridia induced IL-22 production in intestinal epithelial cells, which regulates intestinal permeability and allergen absorption (49). Another study by the same group demonstrated that transferring the microbiome from cow's milk allergic infants and subsequently sensitizing to the major milk allergen β-lactoglobulin (BLG) plus cholera toxin led to increased BLG-specific IgE, IgG1 and reactions on BLG challenge, compared to mice that received microbiome transfers from healthy infants. Supplementation with one specific bacteria from the Clostridia family, *Anaerostipes caccae*, was found to be protective against food allergy, demonstrated by reduced BLG-specific IgE and IgG1 and reduced anaphylaxis on BLG challenge (50). Taken together, data from these studies indicate that manipulating the microbiome could have a therapeutic effect on food allergy (Figure 2).

### Therapies Targeting Siglecs on B Cells and Mast Cells

Inhibitory Siglecs are ITIM-containing sialic acid-binding lectins expressed on a variety of immunologic leukocytes, including B cells and mast cells, making them attractive therapeutic targets. Siglec 2 (CD22) is an inhibitory Siglec on the surface of B cells that can be exploited to induce tolerance through its simultaneous engagement with the B cell receptor (51). An example of this approach for peanut allergy utilized liposomes co-displaying a CD22 ligand and Ara h 2 to successfully prevent production of Ara h 2-specific IgE, rendering the mice tolerant to this major peanut allergen (52). Siglec 3 (CD33) is displayed on the surface of mast cells and can be manipulated through co-ligation of IgE bound to FcεRI to prevent degranulation. Indeed, *in vivo* studies demonstrated the utility of liposomes co-displaying anti-IgE and a CD33 ligand, which completely blocked anaphylaxis on challenge (53). Finally, Siglec 8 is found on human eosinophils and mast cells. Targeting Siglec 8 directly with antibodies shows some promise for inhibiting mast cell degranulation (54). However, liposomes co-displaying Siglec 8 and antigen, which recruits Siglec 8 to the IgE-FcεRI complex, suppresses and desensitizes mast cells (55).

## Discussion

The standard of care for food allergy is avoidance of the offending food and ready access to epinephrine in case of accidental exposure. Although Palforzia, an oral immunotherapy (OIT) drug for the treatment of peanut allergy has been approved by the FDA, it will not be adopted by all food allergy patients due to the frequent allergic side effects, daily dosing and accessibility. Therefore, there is still an urgent need for improved food allergy therapies that will elicit fewer side effects, can be given less frequently, and induce tolerance. As described above, there are many promising candidates for therapies, however the few that have made it into clinical trials have not proven to be as effective as anticipated. These findings demonstrate a disconnect between mouse models of food allergy and human outcomes. In our opinion, identifying a mouse model that can accurately predict responses in humans would be a major breakthrough toward developing therapies. A successful clinical trial with an approach developed in mice will lead to understanding the underlying immunology and biomarkers that are correlated with successful outcomes. In summary, there are a plethora of promising therapeutic approaches being investigated to mitigate the food allergy epidemic.

## Author Contributions

MK and JS equally contributed to reviewing the literature and writing the manuscript. All authors contributed to the article and approved the submitted version.

## Funding

MK is funded by the Department of Defense (W81XWH-16-1-0302 and W81XWH-21-1-0315) and JS was funded by a T32 through the National Institutes of Health (AI007062).

## Conflict of Interest

The authors declare that the research was conducted in the absence of any commercial or financial relationships that could be construed as a potential conflict of interest.

## Publisher's Note

All claims expressed in this article are solely those of the authors and do not necessarily represent those of their affiliated organizations, or those of the publisher, the editors and the reviewers. Any product that may be evaluated in this article, or claim that may be made by its manufacturer, is not guaranteed or endorsed by the publisher.
